# How can artificial intelligence models assist PD-L1 expression scoring in breast cancer: results of multi-institutional ring studies

**DOI:** 10.1038/s41523-021-00268-y

**Published:** 2021-05-26

**Authors:** Xinran Wang, Liang Wang, Hong Bu, Ningning Zhang, Meng Yue, Zhanli Jia, Lijing Cai, Jiankun He, Yanan Wang, Xin Xu, Shengshui Li, Kaiwen Xiao, Kezhou Yan, Kuan Tian, Xiao Han, Junzhou Huang, Jianhua Yao, Yueping Liu

**Affiliations:** 1 Department of Pathology, The Fourth Hospital of Hebei Medical University, Shijiazhuang, Hebei China; 2 AI Lab, Tencent, Shenzhen, Guangdong China; 3 Department of Pathology, West China Center of Medical Sciences, Sichuan University, Chengdu, Sichuan China; 4 Department of Pathology, Affiliated Hospital of Hebei University, Baoding, Hebei China; 5 Department of Pathology, Xingtai People’s Hospital/Hebei Medical University Affiliated Hospital, Xingtai, Hebei China; 6 Department of Pathology, Cangzhou Hospital of Integrated TCM-WM, Cangzhou Hebei, China

**Keywords:** Breast cancer, Tumour immunology

## Abstract

Programmed death ligand-1 (PD-L1) expression is a key biomarker to screen patients for PD-1/PD-L1-targeted immunotherapy. However, a subjective assessment guide on PD-L1 expression of tumor-infiltrating immune cells (IC) scoring is currently adopted in clinical practice with low concordance. Therefore, a repeatable and quantifiable PD-L1 IC scoring method of breast cancer is desirable. In this study, we propose a deep learning-based artificial intelligence-assisted (AI-assisted) model for PD-L1 IC scoring. Three rounds of ring studies (RSs) involving 31 pathologists from 10 hospitals were carried out, using the current guideline in the first two rounds (RS1, RS2) and our AI scoring model in the last round (RS3). A total of 109 PD-L1 (Ventana SP142) immunohistochemistry (IHC) stained images were assessed and the role of the AI-assisted model was evaluated. With the assistance of AI, the scoring concordance across pathologists was boosted to excellent in RS3 (0.950, 95% confidence interval (CI): 0.936–0.962) from moderate in RS1 (0.674, 95% CI: 0.614–0.735) and RS2 (0.736, 95% CI: 0.683–0.789). The 2- and 4-category scoring accuracy were improved by 4.2% (0.959, 95% CI: 0.953–0.964) and 13% (0.815, 95% CI: 0.803–0.827) (*p* < 0.001). The AI results were generally accepted by pathologists with 61% “fully accepted” and 91% “almost accepted”. The proposed AI-assisted method can help pathologists at all levels to improve the PD-L1 assay (SP-142) IC assessment in breast cancer in terms of both accuracy and concordance. The AI tool provides a scheme to standardize the PD-L1 IC scoring in clinical practice.

## Introduction

Breast cancer is one of the most common malignant tumors for women worldwide^1^. Programmed death 1 (PD-1)/programmed death ligand-1 (PD-L1) immunotherapy is one of the most promising treatments for breast cancer, relying on and helping the patient’s immune system to fight cancers^2–4^, and offering a personalized and less invasive alternative therapy. However, only a portion of patients with breast cancer responds to immunotherapy. Nevertheless, the Impassion 130 study indicated clinically meaningful prolonged overall survival for PD-L1 positive patients with tumor-infiltrating immune cell (IC) score greater than 1% when atezolizumab combined with nab-paclitaxel were used as first-line treatment for unresectable local advanced or metastatic triple-negative breast cancer (TNBC)^5^. PD-1/PD-L1 can therefore be used as an effective biomarker to identify patients suitable for immunotherapy^6^. Following clinical trial reports, the U.S. Food and Drug Administration (FDA) has approved Ventana PD-L1 (SP142) as the companion diagnostic tool for PD-L1 immunotherapy^7^. As the main manufacturer of SP142 assays, Roche proposed a guideline for SP142 staining assessment by estimating the IC ratio^8^. However, this scoring guideline is based on description and examples, and is therefore subjective. Moreover, several studies with a broad range of evaluators have shown that pathologists have low rates of agreement and repeatability in assessing PD-L1 expression^9–12^. Consequently, an objective, repeatable, and accurate PD-L1 evaluation method is desirable.

The emergence of digital image analysis is also expected to improve this current situation. The potential of artificial intelligence (AI) technologies such as deep learning algorithms in helping pathologists improve diagnostic accuracy, concordance, and efficiency had been reported^13–18^. Specifically, several AI models have been developed for PD-L1 analysis: for instance, a deep learning model was developed for epithelial cell segmentation in PD-L1 images^19–21^, and a semi-supervised method was proposed for stratification of non-small cell lung carcinoma (NSCLC) for anti-PD-L1 immunotherapy by registering images at different magnifications^22^. Nevertheless, most existing AI models for PD-L1 evaluation have been developed for NSCLC and few applications on breast cancer can be found in the literature.

On the other hand, multi-institutional ring studies are a standard and effective way to evaluate the reproducibility and concordance of a scoring protocol. The blueprint project^11^ compared four PD-L1 assays on NSCLC tumors and evaluated the reliability of PD-L1 scoring. This project recruited 3 pathologists to evaluate 39 specimens in phase I and 18 pathologists to score 81 PD-L1 stained samples in phase II studies. Moreover, a recent ring study^9^ evaluated the concordance of PD-L1 IC scoring on 100 patients with TNBC across 19 pathologists. Ultimately, these reader studies indicate that current PD-L1 scoring protocols suffer from poor reproducibility across multiple pathologists. Furthermore, these studies only evaluated the pathologists’ performances and did not involve AI in the trial.

Advances in AI technology make its adoption imperative in assisting pathologists who score PD-L1 expression. In this investigation, we proposed a deep-learning AI model that quantifies both aggregated and scattered ICs in a unified framework. We organized one of the largest multi-institutional ring studies to establish a PD-L1 evaluation standard with an emphasis on evaluating the role of AI in PD-L1 expression assessment, the acceptance of AI by pathologists, and the limitation of the IC scoring protocol in current clinical practice.

## Results

**Fig. 1:**
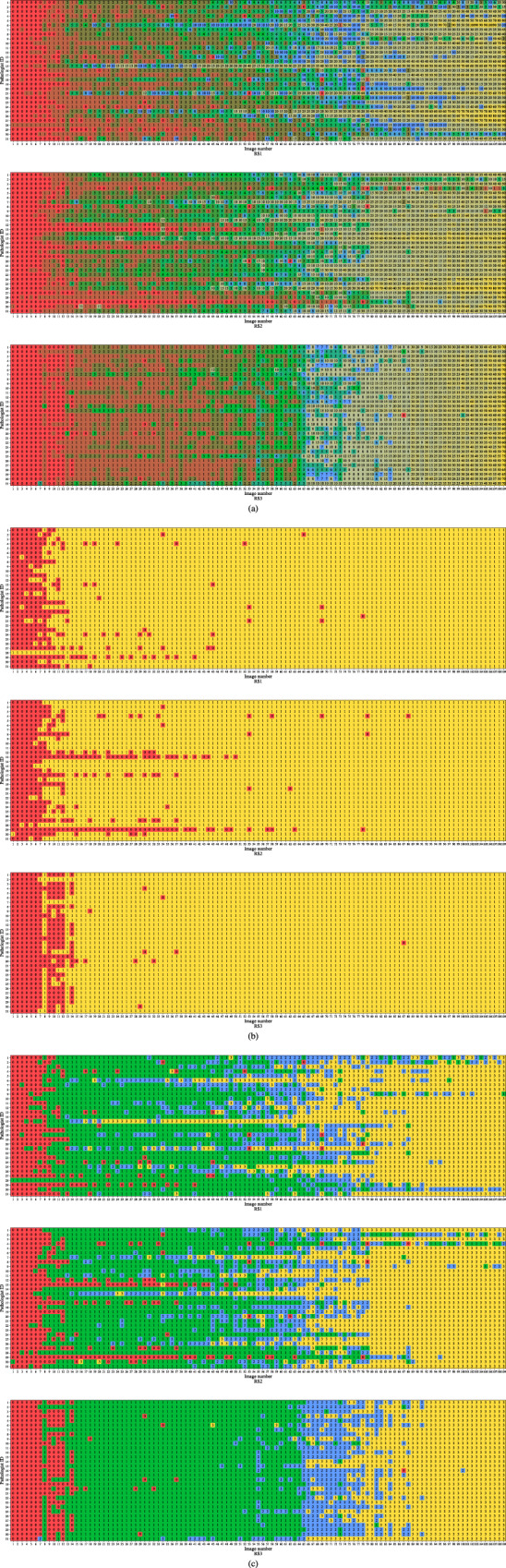
Full IC score from 31 pathologists for 109 images in three-ring studies. **a** Continuous score. **b** 2-category score. The colors red and yellow represent the category scores 1 and 2, respectively. **c** 4-category score. The colors red, green, blue, and yellow represent the category scores 1, 2, 3, and 4, respectively.

In this section, we report the outcomes of the multi-institutional ring study using the following aspects: concordance, accuracy, and acceptance of AI results. The full continuous, 2-category and 4-category PD-L1 (SP142) IC scores evaluated by the 31 pathologists using the 109 test images in the three-ring studies are shown in Fig. 1.

### Results of AI-assisted PD-L1 scoring model

**Fig. 2:**
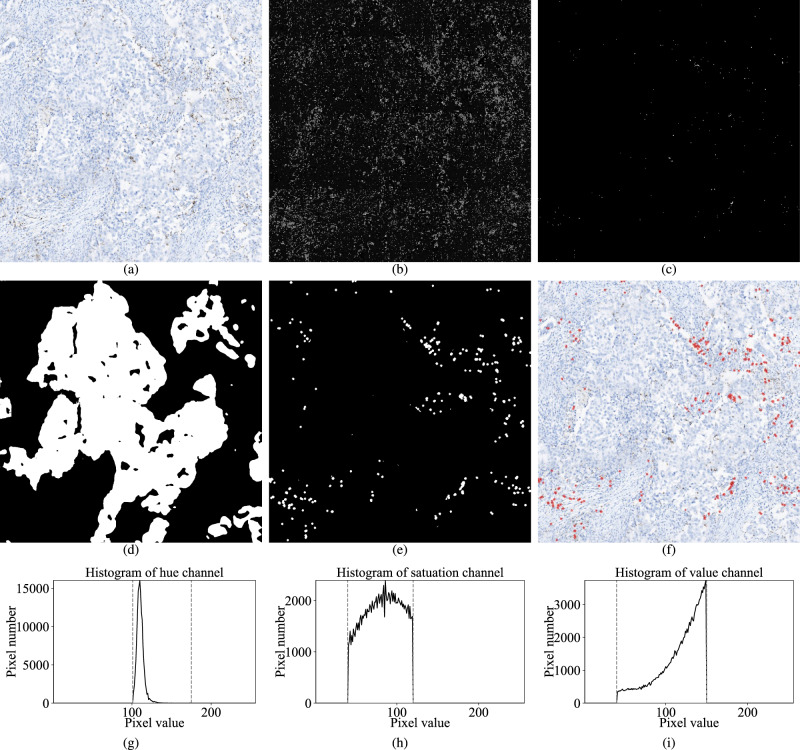
Visual results of our AI-assisted PD-L1 scoring model. **a** Input image of X20 magnification. **b** Hue channel of the transformed image. **c** PD-L1 stained cells mask $M_{\mathrm{stain}}$, including the stained cells inside the epithelial and necrotic regions. **d** Epithelium mask $M_{\mathrm{epithelium}}$. **e** IC mask $M_{\mathrm{IC}}$, which is the result after excluding stained cells inside the epithelial and necrotic regions and then image morphology processing on $M_{\mathrm{stain}}$. **f** Input image overlaid with IC mask $M_{\mathrm{IC}}$. Predicted IC score = 1.7 %. **g**–**i** The histograms of HSV image channels for stained PD-L1 (SP142) IC regions. Two gray dashed lines indicate the threshold on each channel.

The visual results of our AI-assisted PD-L1 scoring model are shown in Fig. 2a–f. We evaluated the performance of the proposed AI-assisted model on the 109 test images using the 2-category and 4-category gold standard scores provided by expert pathologists. The 2-category score accuracy was 0.963 (105/109 images), with an AUC of 0.888 and a 2-class weighted F1 score of 0.962. For the 4-category score, accuracy was 0.752 (82/109 images), with an AUC of 0.797, and 4-class weighted F1 score of 0.764. It can be noticed that four images fail to correct the estimated for the 2-category scoring. One case was underestimated on the stain regions, and three cases were falsely over-estimated on stain regions with the slightly underestimated necrotic region. It indicates that the threshold-based stain region segmentation method has a few optimization potentials. Details of the four cases are shown in Supplementary Fig. 1. Furthermore, the end-to-end epithelium and necrotic region detection neural network models were evaluated by comparing the performance with and without these models. With the epithelium and necrotic region detection, the RMSE of the continuous IC score reduced from 0.10 to 0.05, and the MAE reduced from 0.05 to 0.03. Several examples are shown in Supplementary Fig. 2. In addition, the concordance of the AI model on two different whole slide scanners from different vendors was evaluated. We rescanned all slides used in this study on a different scanner (NanoZoomer S210 Digital slide scanner C13239-01, Hamamatsu Photonics K.K., Japan) at ×40 magnification with the scanning resolution of 0.2285 µm/pixel. Same image patches were selected, resized to the same resolution, and white balanced. The ICC31 was used to evaluate the concordance of AI-predicted IC scores from images of the two scanners. The concordance score was “excellent” (ICC31 = 0.98, 95% CI: 0.97–0.99), which indicates that our AI model generates consistent results on different scanners. Several examples of images from the two scanners can be found in Supplementary Fig. 3.

### Concordance analysis for continuous PD-L1 (SP142) IC scores in each ring study

**Fig. 3:**
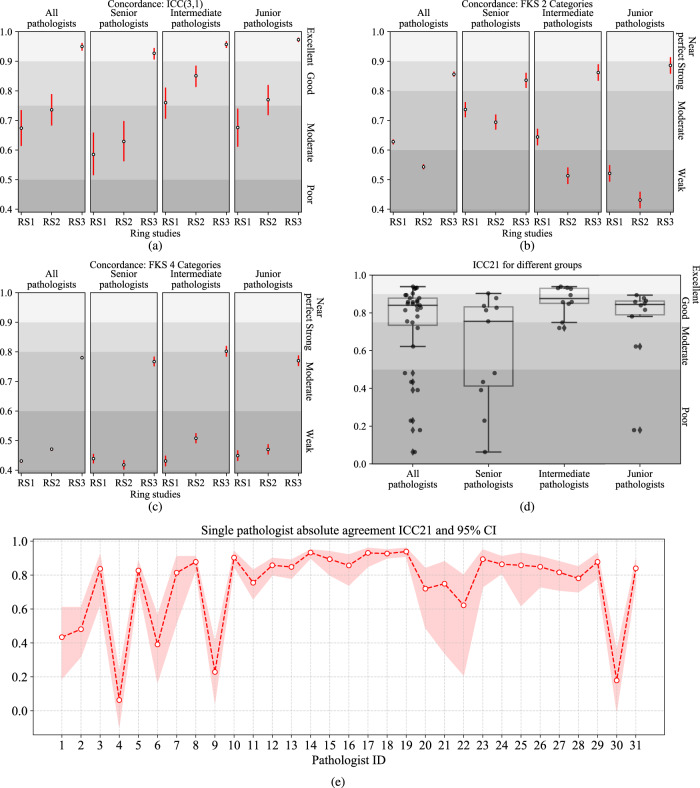
The concordance of IC scoring. The white and black circles indicate the values, and the red bars indicate a 95% confidence interval. **a** Continuous concordances ICC31 in three-ring studies. **b** The FKS concordances for a 2-category score. **c** The FKS concordances for a 4-category score. **d** Boxplots of intra-pathologist concordances ICC21 between RS1 and RS2 for all and three levels of pathologists. The center bar of each box represents the median value, and the box body extends from the 25th to the 75th percentile of values in one group. Black circles indicate the ICC21 of the individual pathologists, and black diamonds indicate the outliers. **e** Intra-pathologist concordances ICC21 for individual pathologists between RS1 and RS2. The shadow area indicates the 95% CI.

The concordance results in each respective ring study are shown in Fig. 3a. ICC31 was 0.674 (95% confidence interval (CI): 0.614–0.735) for RS1, and 0.736 (95% CI: 0.683–0.789) for RS2. Both values were <0.75 and were hence interpreted as “moderate” concordance. With the assistance of the proposed AI model, ICC31 for RS3 improved to 0.95 (95% CI: 0.936–0.962), which is an “excellent” concordance.

Among all the pathologists, the intermediate group outperformed the senior and junior groups in RS1 and RS2 (p < 0.001). However, with the help of the AI-assisted model, the junior group achieved the best ICC31 (0.973, 95% CI: 0.965–0.980, *p* < 0.001) in RS3, and the senior group achieved the largest ICC improvement, from 0.629 (95% CI: 0.562–0.698) in RS2 to 0.927 (95% CI: 0.906–0.945) in RS3 (*p* < 0.001). We found that all three groups benefited from the AI-assisted model when results are compared between RS2 and RS3 (*p* < 0.001).

### Concordance analysis for categorical IC scores in each ring study from all pathologists

Figure 3b, c shows the FKS results for the 2-category and 4-category PD-L1 (SP142) IC scores. For the 2-category score, the concordance was improved to “strong” (0.856, 95% CI: 0.848–0.865) with AI-assisted model in RS3 from “moderate” (0.628, 95% CI: 0.619–0.636) in RS1 (*p* < 0.001), and “weak” (0.543, 95% CI: 0.535–0.552) in RS2 (*p* < 0.001). For the 4-category score, scoring concordance was “moderate” (0.780, 95% CI: 0.775–0.786) in RS3, with a significant improvement (*p* < 0.001) from “weak” in RS1 (0.431, 95% CI: 0.425–0.436) and RS2 (0.471, 95% CI: 0.465–0.476).

### Intra-pathologist concordance

The average intra-pathologist concordance (ICC21) between RS1 and RS2 was 0.737 (95% CI: 0.595–0.819). The ICC21 for each pathologist is shown in Fig. 3e. Average ICC21 scores were 0.601 (95% CI: 0.443–0.709), 0.866 (95% CI: 0.751–0.919), and 0.758 (95% CI: 0.605–0.840) for the senior, intermediate, and junior pathologists, respectively (Fig. 3d).The intermediate group had better intra-pathologist concordance than the senior and junior groups (*p* < 0.001). In addition, the concordance of 2-category IC scoring was evaluated. 5.8% (196 out of 3379) binary scores were different for the same pathologist between RS1 and RS2 (Supplementary Fig. 4). Similarly, we calculated the intra-pathologist concordances between RS1 and RS3, and between RS2 and RS3 (Supplementary Fig. 5) the average ICC21 of RS1–RS3 was 0.756 (95% CI: 0.580–0.845), and the one of RS2-RS3 was 0.784 (95% CI: 0.642–0.858).

### Accuracy evaluation in ring studies

**Fig. 4:**
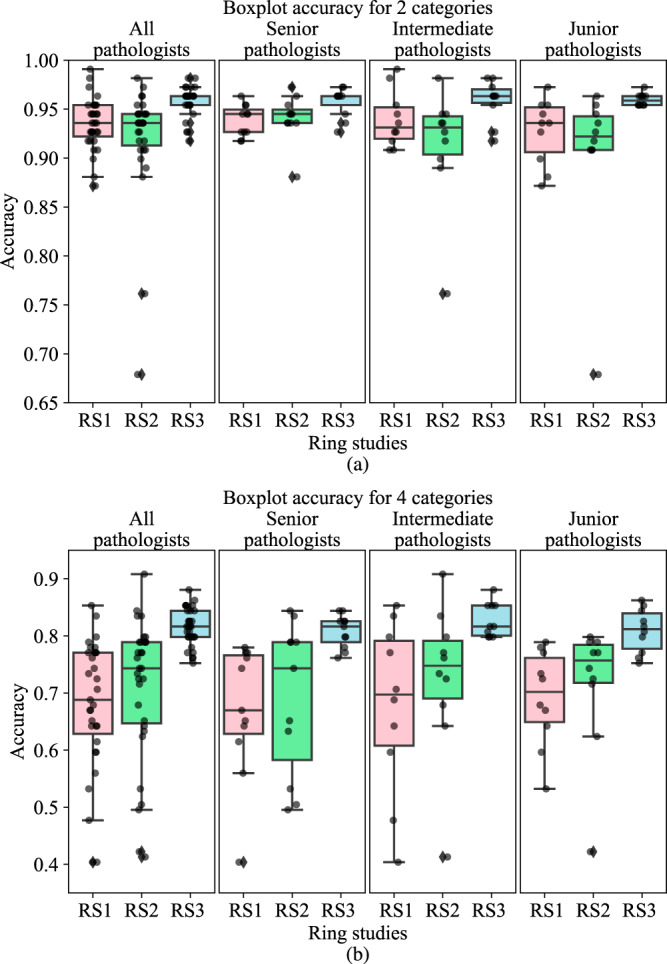
Boxplots of scoring accuracies in three-ring studies for pathologists in different levels. The center bar of each box represents the median value, and the box body extends from the 25th to the 75th percentile of values in one ring study. Black circles indicate the accuracy of the individual pathologists, and black diamonds indicate the outliers. **a** 2-category score. **b** 4-category score.

For the 2-category scoring (Fig. 4a), pathologists alone achieved relatively high performance in terms of average accuracy in RS1 and RS2, at 0.935 (95% CI: 0.926–0.945) and 0.92 (95% CI: 0.899–0.942), respectively. AI assistance gave a significant accuracy boost (*p* < 0.001) in RS3 (0.959, 95% CI: 0.953–0.964), which improved 4.2% from RS2. For 4-category scoring (Fig. 4b), the average scoring accuracy also had a significant improvement (*p* < 0.001) of 14.7% from RS2 to RS3 through AI assistance, at 0.815 (95% CI: 0.803–0.827) in RS3 vs. 0.710 (95% CI: 0.665–0.756) in RS2.

Despite having different levels of experience, the pathologists showed comparable scoring accuracy, especially after the AI- assistance. For 2-category scoring, the senior, intermediate, and junior groups had accuracy improvements of 1.7% (*p* < 0.001), 4.9% (*p* < 0.001), and 6.1% (*p* < 0.001) from RS2 to RS3, respectively. Similarly, for 4-category scoring, the senior, intermediate, and junior groups had improvements of 16.9% (*p* < 0.001), 13.9% (*p* < 0.001), and 13.1% (*p* < 0.001) from RS2 to RS3, respectively.

Figure 4 also shows that pathologists in the different experience groups demonstrated very similar accuracy in RS3 despite their performances having varied greatly in RS1 and RS2, both within and across groups (Supplementary Table 1).

### Acceptance of AI results

**Fig. 5:**
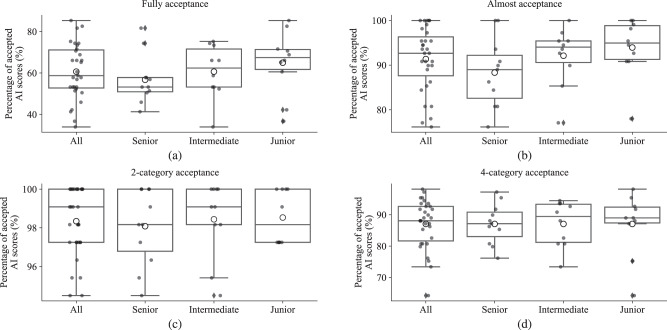
Boxplots of acceptance of continuous and categorial AI scores. The center bar of each box represents the median value, and the box body extends from the 25th to the 75th percentile of values in one group. Black circles indicate the value of individual pathologists, and black diamonds indicate the outliers. The larger black circles with white inside indicate the average value of one group. **a** “Fully accepted” of continuous AI score with a scoring difference <1%. **b** “Almost Acceptance” of continuous AI score with scoring difference < 5%. **c** Acceptance of 2-category AI score. **d** Acceptance of 4-category AI score.

In this section, we analyze the difference between the pathologists’ IC scores and the reference AI scores in RS3 to evaluate the acceptance of AI results by the pathologists. We considered that the AI score is “fully accepted” by a pathologist if the score difference is smaller than 1% absolute value (Fig. 5a) and “almost accepted” if smaller than 5% (Fig. 5b), excluding AI scores that change score categories. We also considered that the AI score is “categorically accepted” if the pathologist’s score and the AI score are in the same category (Fig. 5c, d).

As shown in Fig. 5a, 60.6% of the AI scores were “fully accepted” by pathologists on all images, with senior pathologists at 56.7%, intermediate pathologists at 60.6%, and junior pathologists at 65.0%. When considering “almost acceptance” by all pathologists, the rate improved to 91.4%.

The acceptance of the 2- and 4-category AI scores are illustrated in Fig. 5c, d, respectively. For the 2-category scores, on average, all pathologists, senior, intermediate, and junior groups had 98.3%, 98.1%, 98.4%, and 98.5% similarity in category scores as the AI, respectively. For the 4-category, all pathologists and the three groups had 87.1% similarity in category score as the AI on average, whereas the junior group had smaller acceptance variability than the other two groups.

## Discussion

PD-L1 is an important biomarker whose accurate assessment is essential in cancer patient triage for immunotherapy. Drug companies and pathologist associations have proposed several interpretation guidelines targeted at reliable and consistent PD-L1 expression assessment. However, several reader studies^9,11^ have shown that reproducibility and concordance are poor across pathologists. Pathologists are good at identifying and locating cancer regions, but are not efficient at counting and computing, which are the strengths of AI models. We speculate that combining the strengths of pathologists and AI models by providing pathologists with easily perceived AI counting results is the way to improve pathologists’ scoring reliability. To validate this concept, we conducted one of the largest reader studies for PD-L1 expression scoring. The Blueprint study recruited 18 pathologists evaluating 81 specimens of lung cancer and Reisenbichler’s study recruited 19 pathologists reading 100 breast cancer cases^9,11^. In the present study, we organized 31 pathologists with various experience levels to score 109 PD-L1 stained breast cancer images. Furthermore, we proposed an AI-assisted scoring algorithm based on deep-learning methods to help pathologists with the PD-L1 IC scoring. To the best of our knowledge, this study involved specifically AI method in PD-L1 breast cancer evaluation. In addition to the consistency and accuracy evaluation similar to most other reader studies, our ring study had been designed to answer the following questions: (1) what is AI’s role in PD-L1 expression assessment? (2) to what extent can AI models help pathologists of various experience levels? (3) how much do pathologists trust and accept AI? and (4) what is the best practical scoring scheme for PD-L1 expression in breast cancer?

What is AI’s role in PD-L1 expression assessment? Human pathologists are not good at precisely evaluating ratios, especially when hundreds of cells are presented. Therefore, Roche’s guide and the Impassion130 protocol used 1% of PD-L1 (SP142) IC expression to stratify patients into two groups; they demonstrated a prolonged overall survival rate for PD-L1 (SP142)-positive patients when IC score is >1%^8^. The 2-category scoring scheme served the purpose of a rough but reliable assessment. Our study demonstrated that the 2-category concordance across pathologists was merely 0.628 since borderline cases (those around 1% IC) are hard to be reliably differentiated by pathologists. Binary stratification can decide whether a patient should receive immunotherapy, but this is not sufficient for personalized therapy, especially since patients with different levels of PD-L1 expression may need to receive different therapeutic plans in terms of drug dose and therapy length. Some difficult cases, especially those with PD-L1 scores around 1%, might be misinterpreted by pathologists, and thus result in the misclassification of patients. In routine diagnosis, pathologists score PD-L1 as negative by completely relying on their visual perception. Positive patients misclassified as negative could be deprived of the benefits of immunotherapy, whereas negative patients scored as positive could be exposed to unnecessary, costly, and potentially toxic therapy. Because of these limitations, the 4-category scoring scheme was used in our study, which allowed for more granular patient stratification. However, this scheme also increased scoring difficulty and lowered the concordance, which was merely 0.471 in RS2. On the other hand, AI-assisted scores gave pathologists a ballpark estimation, making them more confident in providing multi-category scores. Our study demonstrated that the 2-category concordance was boosted by 0.228 and the 4-category concordance by 0.309 with AI assistance. The improved ICC in RS3 also suggested that in addition to a defined guideline, the AI-assisted diagnosis model with quantified reference feedback might be a valuable tool for pathologists to standardize the process of PD-L1 expression assessment in breast cancer. Our experiments were conducted on selected regions from a WSI with the manual exclusion of artifactual staining regions, since we wanted to ensure that all pathologists read the same content in the regions containing mostly the epithelium, interstitial, and stained regions. However, region selection is also part of the scoring process and should have been evaluated separately. In daily practice, the pathologists have full control of the workflow, and the algorithm just provides the tedious computation part. Our experimental setup reflected the daily practice and the AI model relieved the pathologists from the uncertain and tedious counting work. Moreover, different tiles from the same WSI could be selected if the pathologists worked independently. We conducted an experiment evaluating two individual pathologists’ tile selection on 20 WSIs. Three tiles were selected from each WSI. With the assistance of the AI models, two pathologists scored their tiles and the average IC score of each WSI. The ICC31 was computed to compare the IC score results by the two pathologists, which was regarded as “excellent” (ICC31 = 0.967, 95% CI: 0.92–0.99). Details can be found in Supplementary Fig. 6. Based on our results, AI models can have a big role in helping pathologists evaluate borderline cases and provide assistance in more granular scoring for personalized treatment. Moreover, applying this approach to routine diagnostic practice could improve the diagnostic efficiency of pathologists, and relieve the pressure especially from those who lack more experience.

To what extent can AI models help pathologists of various experience levels? In our study, the pathologists had been divided into three levels according to their level of experience. These groups of different levels demonstrated different performances in RS1 and RS2. For instance, intermediate pathologists had the best concordance in continuous scoring and 4-category scoring, possibly because they have a good balance of reading experience (good for senior pathologists) and counting capability (good for junior pathologists). Senior pathologists were more confident and their own habits in diagnosis may not be easily influenced. They performed best in 2-category scoring (FKS: 0.694 vs. 0.513 and 0.431), where the cutoff value of 1% has been proven meaningful in clinical trials. However, with the assistance of AI in RS3, all pathologists boosted their performances and the gaps among the different levels became smaller. These findings indicate that AI can not only help improve the consistency of pathologists with various experience levels, but also help level the playing field, closing the experience gaps across pathologists.

How much do pathologists trust and accept AI? Our AI model detected and marked the PD-L1 stained ICs in the intratumoral and peritumoral stroma, and also computed the IC score based on the Roche guide. These AI results had been provided to pathologists as references in RS3. If a pathologist trusted the AI result, he/she will take the AI score with little or no modification; otherwise, the pathologist will adjust the AI score according to their perception on whether the AI over-computed or under-computed the IC score. Results from RS3 indicate that pathologists generally trust AI, with only 7% of all AI scores adjusted by more than 5%. As we have observed, the AI model is not 100% accurate. It could miss weakly stained cells or mistakenly recognize artefacts as stained cells. Epithelial segmentation could also have errors. Furthermore, our AI model can not reliably choose the right regions for scoring. At their current development stage, AI models are not designed to replace pathologists, but rather to assist pathologists. Therefore, it is important that AI results are presented in an intuitive way, so that pathologists can quickly grasp the meaning and make judgments based on these AI scores. We need to combine the strengths of the pathologists and AI models. A pathologist with the assistance of AI is better than a pathologist alone.

What is the best practical scoring scheme for PD-L1 expression in breast cancer? PD-L1 expression scoring currently plays a significant role in the immunotherapy for an increasing number of advanced carcinomas^23–25^. Nevertheless, standardization of this subjective testing has not been achieved. With the AI-assisted model, we tried to establish a PD-L1 evaluation standard in breast cancer and improve the current situation of poor consistency in PD-L1 scoring among pathologists due to subjective assessment or lack of experience. Moreover, the insights from this study may also be applicable to the standardization of other diagnostic biomarkers in other carcinomas. Our findings show that the consistency of both 2-category and 4-category scoring significantly improved. At present, the current clinical studies suggest prolonged overall survival for PD-L1-positive patients with IC scores >1% in breast cancer^8^. However, with the development of clinical trials and the increasingly prominent role of precision medicine, the cutoff value of PD-L1 expression should be optimized to accommodate the benefits of immunotherapy for a variety of patients. Therefore, a more granular classification and more accurate interpretation of PD-L1 scoring could be a trend that would provide the basis for patients to obtain personalized treatment and an accurate therapeutic schedule. With the fine-grained estimated IC score, our proposed method could work with various cutoff values from different clinical applications and trials and could even propose a reliable cutoff value for precision medicine.

The PD-L1 (SP142) IC scoring guide is currently adopted in clinical practice. This guide illustrates two IC patterns, aggregated and scattered, and suggests different strategies for scoring each. For the aggregated pattern, a polygon enclosing the aggregation is delineated for the IC area. For the scattered pattern, a few matching templates are provided for a few concrete scores. Therefore, it is difficult for pathologists to come up with a score when both patterns are presented in one image. Hence, the number of ICs instead of the areas of ICs may be a better indicator of PD-L1 expression, in which case the space between ICs will not be a factor and the two patterns can be processed in the same way. Currently, our AI model handles this problem with a unified framework, where scattered cells are first detected and then the spaces between aggregated cells are closed using morphological operations. Our model is straightforward in using the number of IC cells instead, such as the tumor proportion score (TPS) scoring in lung cancer. PD-L1 evaluation is subjective and to a great extent relies on the experience of the pathologists. In our study, a quantifiable method made the objective evaluation of PD-L1 possible. The proposed AI model could assist pathologists in overcoming the confusing “aggregated” and “scattered” patterns in the Roche guide.

However, this study has several potential limitations. The AI model had been developed and trained following the Roche guide and a few examples of 2-category scoring. The guide had been based on the results of a previously reported clinical trial, and hence could be subject to change after future new trials. Moreover, the gold standard scores used in our study had been based on consensus reading from three experienced pathologists, which are still somewhat subjective. More rigorous gold standard scores based on manual annotation of all PD-L1 stained ICs may be necessary to evaluate the true performance of AI models and pathologists. Also, tiles with non-tumor regions such as cancer in situ, normal areas, and non-specific staining, etc., were manually excluded in our study. Those regions can be detected by our region detection methods that have been developed after the experiment and can be integrated in the future. Besides, the concordance analysis and the P-values obtained from the 109 images in our study show a preliminary tendency that the AI-assisted model could be helpful for the IC scoring of pathologists. However, it is reasonable that more cases will certainly increase the statistical power. Furthermore, although all three ring studies have been conducted on the same online system. The pathologists used different devices, such as personal computer, laptop, cell phone, or tablet computer, to access the online system, which may have contributed to part of the scoring disparity. This factor was not evaluated in our study.

In conclusion, we have developed an AI-assisted model for the quantitative calculation of PD-L1 staining on IC. A multi-institutional ring study demonstrated that AI-assisted scoring could help pathologists improve in PD-L1 assay (SP-142) assessment in terms of both accuracy and concordance. The results show that pathologists of all experience levels could benefit from the AI-assisted model, and that the AI results are generally accepted by pathologists.

## Methods

### Patient cohort and data preparation

One hundred tumor resection samples (formalin-fixed, paraffin-embedded blocks) from 100 patients with invasive breast cancer were collected in the fourth hospital of Hebei Medical University from January to June 2019. Patient characteristics are listed in Table 1. For each block, 4 μm sections were cut using the LEICA RM2255 slicer. These were baked on the TKY-TKA spreader at 65 °C for 1 h and then stained with PD-L1 at a dilution of 1:50 (clone SP142, Ventana Medical Systems, Tucson, USA) using the OptiView DAB IHC detection kit, strictly following the manufacturer’s instructions on benchmark XT automatic immunohistochemistry (IHC) (BenchMark ULTRA, Ventana, Tucson, USA).

**Table 1:** Patient characteristics of 100 cases of invasive breast cancer.

Characteristics	Case number
Age	
≤50	39
>50	61
Histologic type	
Invasive carcinoma of no special type	97
Invasive lobular carcinoma	2
Metaplastic carcinoma	1
Histological grade	
I	3
II	37
III	60
Clinical stage	
I	28
II	57
III	15
IV	0

All immunostained slides were scanned using the Unic digital scanner (precision 600 Series, Unic Technologies, INC. Beijing, China) at ×40 magnification. The data was prepared in the following steps. Firstly, from the scanned whole slide images (WSI), two pathologists manually identified the tumor regions. They also annotated the necrosis area, cancer in situ, and normal areas by strictly following the scoring guideline of Ventana PD-L1 (SP142) in breast cancer provided by Roche guide^8^. Secondly, from the tumor regions, sliding windows with no overlap were scanned through the tumor regions and generated 4246 image patches. The image size was 3290 × 3290 at 0.344 µm/pixel, and resized to 2160 × 2160 as 0.524 µm/pixel, which was approximate to ×20 objective magnification of a normal microscope. After that, images with manually identified necrosis area, cancer in situ, and normal areas were excluded, and 2395 image patches remained. Then, considering the workload, 109 image patches were randomly proposed from the 2395 image patches, with the criteria that the proposed patches should not have non-specific staining, focal contamination, and folding, and should not be similar to other patches in the set. At last, the selected 109 image patches were used for this study. The STARD flow diagram is shown in Supplementary Fig. 7. The images were then uploaded onto an online system for reviewing and scoring.

### Ethics statement

All tissues and data were retrieved under the permission of the institutional research ethics board of the Fourth Hospital of Hebei Medical University with the declaration number of 2020KY112 on 24 February 2020, since it did not involve interaction with human subjects and/or use of individual’s personal identifying information. Informed consent was not required for the use of existing pathological materials with no reveal of identifiable patient information.

### Pathologist recruitment

We organized a multi-institutional ring study for Ventana PD-L1 SP142 assay assessment in invasive breast cancer, recruiting 31 board-certified pathologists from 10 provincial and municipal hospitals. The pathologists were divided into three groups according to their experience: senior (≥10 years, 11 pathologists), intermediate (≥5 years but <10 years, 10 pathologists), and junior (≥2 years but <5 years, 10 pathologists). All pathologists attended training sessions on the Roche PD-L1 scoring guideline.

### Ring study design

The ring studies comprised three rounds of experiments. The purpose of ring study 1 (RS1) was to evaluate the scoring concordance across pathologists in the current clinical practice. Pathologists provided PD-L1 IC scores through an online system after receiving training on the Roche PD-L1 (SP142) IC scoring guideline. During RS1, the pathologists logged onto an online website developed by our team (Supplementary Fig. 8), viewed the images, estimated the area of stained ICs, provided the IC score, and entered their assessments on each of the 109 image patches. After a 2-week washout period, ring study 2 (RS2) was carried out, with the purpose of evaluating intra- and inter-observer concordance. The pathologists performed scoring in the same manner as in RS1. After another 2 weeks of washout period, ring study 3 (RS3) was conducted. Here, the emphasis was on evaluating the role of AI assistance in PD-L1 scoring. The pathologists performed the scoring once again using the same online system, but this time with the assistance of IC score results from our AI model (Supplementary Fig. 9). PD-L1 stained areas identified by our AI model (described in the “AI-assisted IC scoring model” section) and the associated IC scores were provided to the pathologists as reference. The pathologists had the option to adjust the score based on the comparison between the perceived stained areas and AI detected areas. In all three ring studies, the pathologists viewed and scored the images independently and made use of the same online systems. The images were randomly reordered in each ring study.

### IC scoring protocol

According to the Roche guideline, the IC score is defined as the areas of PD-L1 stained IC (of any staining intensity) over the tumor area, which are occupied by tumor cells and associated intratumoral and contiguous peritumoral stroma^8^. In all three ring studies, the pathologists provided continuous IC scores, ranging from 0 to 100%. In the Roche guideline, the 2-category score (<1% IC and ≥1% IC) was used to stratify patients for immunotherapy. In addition, a 4-category score, i.e., at (0%, 1%),(1%, 5%),(5%, 10%), and (10%, 100%) intervals, was proposed in another report^26^ for a more granular stratification. Both categorical score schemes were evaluated in the ring studies.

The gold standard for the categorical PD-L1 (SP142) IC scores of the test images were provided through consensus reading from two experienced pathologists who received formal training from Roche Diagnostics and practiced PD-L1 (SP142) expression scoring in their routine clinical work. Scoring disagreement between the two pathologists was resolved by a third senior pathologist who joined the discussion to reach a consensus. All these three pathologists were not involved in the ring studies.

### Evaluation metrics and statistical analyses

Both score concordance and accuracy were evaluated in the three-ring studies. The two-way mixed-effects intraclass correlation coefficient model of consistency definition with single measurement (ICC31) was adopted for the analysis of continuous IC score concordance^27^. The concordance was regarded as “poor,” “moderate,” “good,” and “excellent” for the ICC values in (0, 0.5), (0.5, 0.75), (0.75, 0.9), and (0.9,1.0), respectively^11,27^. The Fleiss’ kappa statistic (FKS) was applied for the concordance analysis on the 2- and 4-category PD-L1 (SP142) IC scores^28,29^. FKS is an extension of Cohen’s kappa for three raters or more^30^. The FKS can be interpreted as “weak,” “moderate,” “strong,” and “near perfect” for its value in (0.4, 0.6), (0.6, 0.8), (0.8, 0.9), and (0.9, 1), respectively^11,31^. Furthermore, intra-pathologist scoring concordance between RS1 and RS2 was evaluated using a two-way random effect absolute agreement model (denoted as ICC21)^27^. The accuracy evaluation was represented by several metrics, including accuracy, area under the curve (AUC), and weighted F1 score^32^. The evaluation and statistical analyses were performed using Python programming language version 3.6.5, with the Scikit-learn version 0.23.1 and Pingouin version 0.3.3 packages.

### AI-assisted IC scoring model

**Fig. 6:**
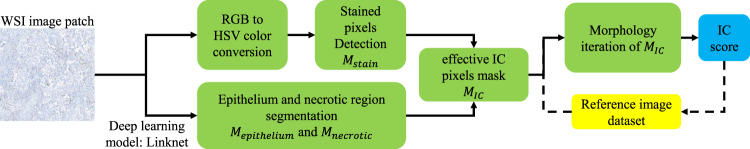
Overall pipeline of the AI-assisted model. The AI model had two parallel threads: the first thread detected and segmented all staining cells; the second thread segmented tumor epithelium and necrotic regions. The ICs were obtained by combining the results of the two threads.

According to Roche’s interpretation guide for Ventana PD-L1 (SP142) expression in patients with TNBC, IC are presented in the intratumoral and contiguous peritumoral stroma that include lymphocytes, macrophages, dendritic cells, and granulocytes. IC score is considered as the proportion of tumor area that is occupied by PD-L1 staining IC of any intensity. Therefore, we designed an AI-assisted PD-L1 IC scoring method following this guideline, as outlined in Fig. 6. Due to the non-specificity of PD-L1 staining, both tumor cells in epithelial regions and ICs in intratumoral and peritumoral stoma could be stained. The AI model had two parallel threads to separately handle stain and tumor detections. The first thread detected and segmented all staining cells of any intensity. The second thread was an end-to-end network that segmented tumor epithelium and necrotic regions. The results from the two threads were combined so that stained cells in epithelial and necrotic regions can be excluded, leaving only ICs in the intratumoral and contiguous peritumoral stroma regions.

### PD-L1-stained cell detection and segmentation

We discovered that the PD-L1-stained cells can be better distinguished from other cells in the hue property than in the original red, green, blue (RGB) properties^33^. Therefore, we first transformed the image from the RGB color space to the hue, saturation, value (HSV) color space and then applied thresholds on the HSV space to obtain the initial detection^34^.

The thresholds were determined using training data from analysis of the HSV histogram of PD-L1 stained cells, which are $\left[ {h_1,h_2} \right]$, $\left[ {s_1,s_2} \right]$, and $\left[ {v_1,v_2} \right]$ for the hue, saturation, and value channels, respectively. The training data consisted of 25 image patches from the Roche guide^8^, with IC scores of 0% (2 images), <1% (6 images), >1% (12 images), 2% (2 images), 5% (1 image), 15% (1 image), and 25% (1 image). The stained IC pixels of 25 Roche images^8^ were used to determine the threshold. The RGB pixel values were converted to HSV values and the histogram of the stained IC regions in the training images were plotted in H, S, V channels, respectively (shown in Fig. 2g–i). By analyzing the histogram to include 95% of all stained IC pixels, we obtained the threshold of $\left[ {h_1,h_2} \right]$, $\left[ {s_1,s_2} \right]$, and $\left[ {v_1,v_2} \right]$, which were [101, 175], [40, 120], and, [40, 150], respectively. By applying these thresholds, a binary mask of stained pixels *M*_stain_ can be obtained, with the pixel value 1 representing the PD-L1 stained pixels.

We then applied image morphological (opening and dilation) operations on *M*_stain_ to smooth out the noise. First, a morphological erosion was applied on the image with a 3 × 3 kernel, which removed small image noise. Then, a dilation operation with a 3 × 3 kernel was adopted to restore the stained regions.

### Epithelium and necrotic region detection

We trained an end-to-end deep learning model (Linknet) to segment the epithelial regions^35^. Linknet is a pixel-wise semantic segmentation network based on an encoder-decoder architecture. The model for the epithelial region segmentation was trained using 2,767 IHC image patches from 41 estrogen receptor, 37 progesterone receptor, and 394 Ki67 WSIs. The necrotic region segmentation model was trained using 2079 image patches from 255 PDL1 (SP142) WSIs. All the image patches were 832 × 832 pixels with 0.848 µm/pixel. The epithelial and necrotic regions were manually annotated on the image patches. The models were trained by nearly 300 epochs by minimizing the mean square loss. The drop rate was r = 0.8, the learning rate was 10^–2^ initially and decreased to 10^–5^ gradually, and the batch size was 64. Image augmentations of random flip and rotation were applied. The models were implemented by Python 3.6, Tensorflow 1.14, and Cuda 10.0 with NVIDIA Tesla P40 GPU (RAM 24 G), with details in ref. ^36^. As a result, a binary mask representing the epithelial region *M*_epithelium_ was predicted from the deep learning model. Similarly, we detected the necrotic region mask *M*_necrotic_.

### Scoring

The effective PD-L1 (SP142) IC pixels mask *M*_IC_ was then obtained by calculating the intersection of *M*_stain_ and the inverse of *M*_epithelium_ + *M*_necrotic_:

1 $$M_{\mathrm{IC}} = M_{\mathrm{stain}} \cap \left[ {{\mathrm{Inv}}\left( {M_{\mathrm{epithelium}} \cup M_{\mathrm{necrotic}}} \right)} \right]$$

where the *M*_IC_ is a binary image with pixel values of 0 and 1, and Inc (·) calculates the inverse binary mask.

The effective IC mask *M*_IC_ located the position of stained ICs in the intratumoral and contiguous peritumoral stroma regions. Furthermore, in the Roche guide there were two types of stained IC regions: aggregated and scattered (Supplementary Fig. 10). In the aggregated case, the enclosed area of the aggregation was treated as the PD-L1 (SP142) IC region. Therefore, we iteratively dilated *M*_IC_ to fill up the space between stained cells. The number of iterations was chosen so that the effective PD-L1 (SP142) IC area matched the IC area in the reference images. The PD-L1 (SP142) IC score was then computed as the ratio of the area of stained PD-L1 (SP142) ICs over the area of effective tumor region:

2 $${\mathrm{IC}} \cdot {\mathrm{score}} = \frac{{\mathop {\sum }\nolimits_{i = 1}^N {\mathrm{Dilation}}\left[ {M_{\mathrm{IC}}} \right] = 1}}{{{N}}},$$

where $\mathop {\sum}\nolimits_{{\mathrm{i}} = 1}^{{N}} {\left( \cdot \right)}$ calculates the pixel number matching the condition of $\mathop {\sum }\nolimits_{i = 1}^N {\mathrm{Dilation}}\left[ {M_{\mathrm{IC}}} \right] = 1$, *N* is the total pixel number of mask *M*_IC_.

### Reporting summary

Further information on research design is available in the Nature Research Reporting Summary linked to this article.

## Supplementary information

Supplementary Information

Reporting Summary

## Data Availability

All data files associated with this study are openly available from the following data record 10.6084/m9.figshare.14363486^37^.

## Code availability

The code can be used only for “non-commercial” purpose and under the permission of the correspond author. The code for analysis and experiments were publicly available here https://github.com/LWCHN/PDL1_SP142_Breast_IC_score.
