# Supplementary material for: How can artificial intelligence models assist PD-L1 expression scoring in breast cancer: results of multi-institutional ring studies
Source: NPJ Breast Cancer. 2021 May 26;7:61. doi: 10.1038/s41523-021-00268-y (PMC8155065; doi:10.1038/s41523-021-00268-y)
Supplement: Supplementary file 1 — Supplementary Information [file 41523_2021_268_MOESM1_ESM.pdf]

Supplementary Information: How Can Artificial Intelligence Models Assist PD-L1 Expression Scoring in Breast Cancer: Results of Multi-institutional Ring Studies

Xinran Wang[1,#], Liang Wang[2,#], Hong Bu[3], Ningning Zhang[1], Meng Yue[1], Zhanli Jia[1], Lijing Cai[1], Jiankun He[1], Yanan Wang[4], Xin Xu[5], Shengshui Li[6], Kaiwen Xiao[2], Kezhou Yan[2], Kuan Tian[2], Xiao Han[2], Junzhou Huang[2], Jianhua Yao[2,*], Yueping Liu[1,*]

[1]*Department of Pathology, The Fourth Hospital of Hebei Medical University, Shijiazhuang, Hebei, China*
[2]*AI Lab, Tencent, Shenzhen, Guangdong, China*
[3]*Department of Pathology, West China Center of Medical Sciences, Sichuan University, Chengdu, Sichuan, China*
[4]*Department of Pathology, Affiliated Hospital of Hebei University, Baoding, Hebei, China*
[5]*Department of Pathology, Xingtai People's Hospital/Hebei Medical University Affiliated Hospital, Xingtai, Hebei, China*
[6]*Department of Pathology, Cangzhou Hospital of Integrated TCM-WM, Cangzhou, Hebei, China*

* Corresponding Authors:
Yueping Liu, Department of Pathology, The Fourth Hospital of Hebei Medical University, No. 12 Jiankang Road, Shijiazhuang 050011, China. Phone: 86-311-86095374. Email: annama@163.com; and Jianhua Yao, Tencent AI Lab, Tencent Binhai Building, No. 33, Haitian Second Road, Nanshan District, Shenzhen,518054, China. Phone: 86-755-86013388. Email: jianhuayao@tencent.com

# Xinran Wang and Liang Wang contributed equally to this article.

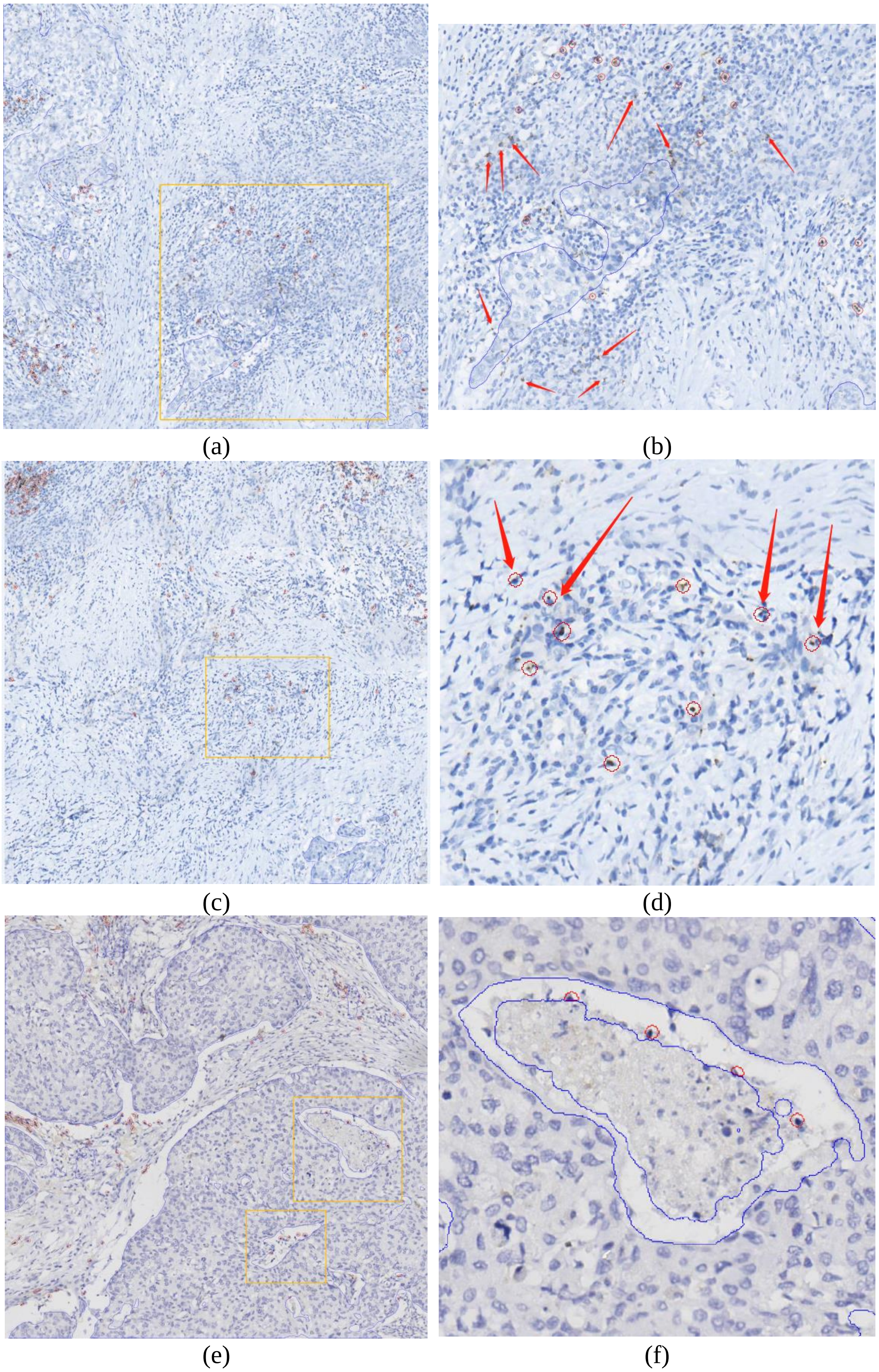
(a)
(b)
(c)
(d)
(e)
(f)

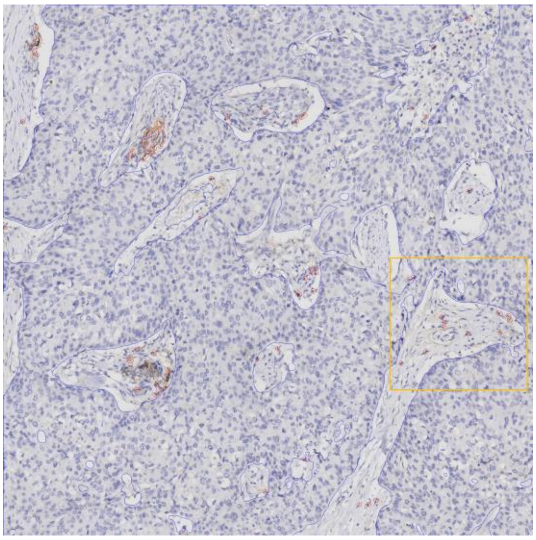

(g)

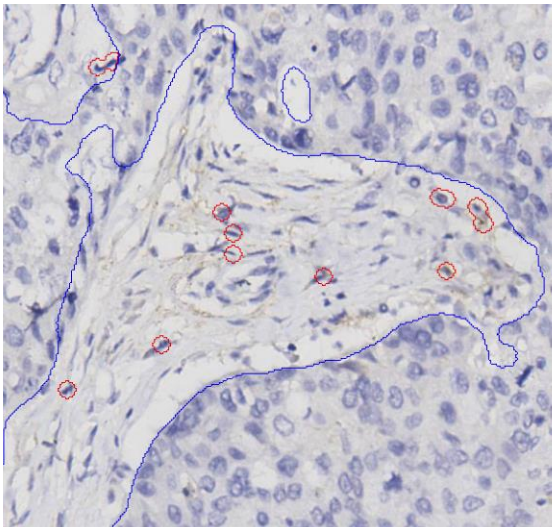

(h)

Supplementary Figure 1: Four cases fail to be correctly estimated for the 2-category scoring by AI model only. (a) Failed case 1: Predicted IC score = 0.5%, Ground truth IC score = 1.0%. The average and standard deviation of IC scores on this case from the pathologists was 1.81%±1.27% in RS1 and 1.80%±1.40% in RS2. (b) ROI of (a). Several stained regions were not detected (indicated by red arrows), which could be the main reason of the underestimated IC score of the AI assisted model. (c) Failed case 2: Predicted IC score = 1.3%, Ground truth IC score < 1.0%. The average and standard deviation of IC scores on this case from the pathologists was 1.84%±1.12% in RS1 and 2.16%±1.95% in RS2. (d) ROI of (c). From the zoomed-in ROI, the stained colors of some regions were rather weak (indicated by red arrows). These over-estimated regions elevated the predicted IC score. (e) Failed case 3: Predicted IC score = 1.3%, Ground truth IC score < 1.0%. The average and standard deviation of IC scores on this case from the pathologists was 0.53%±0.64% in RS1 and 0.57%±0.59% in RS2. (f) ROI of (e). The white space region between the detected necrosis and tumor regions is detected as interstitial region. The stained colors of the detected IC region in the ROIs are weak. This makes the IC score be over-estimated. (g) Failed case 4: Predicted IC score = 1.1%, Ground truth IC score < 1.0%. The average and standard deviation of IC scores on this case from the pathologists was 1.69%±1.19% in RS1 and 1.69%±1.03% in RS2. (h) ROI of (g). Several blue regions in the zoomed ROI are observed, which could be considered as false over-estimated stain regions and lead the predicted IC score greater than 1%.

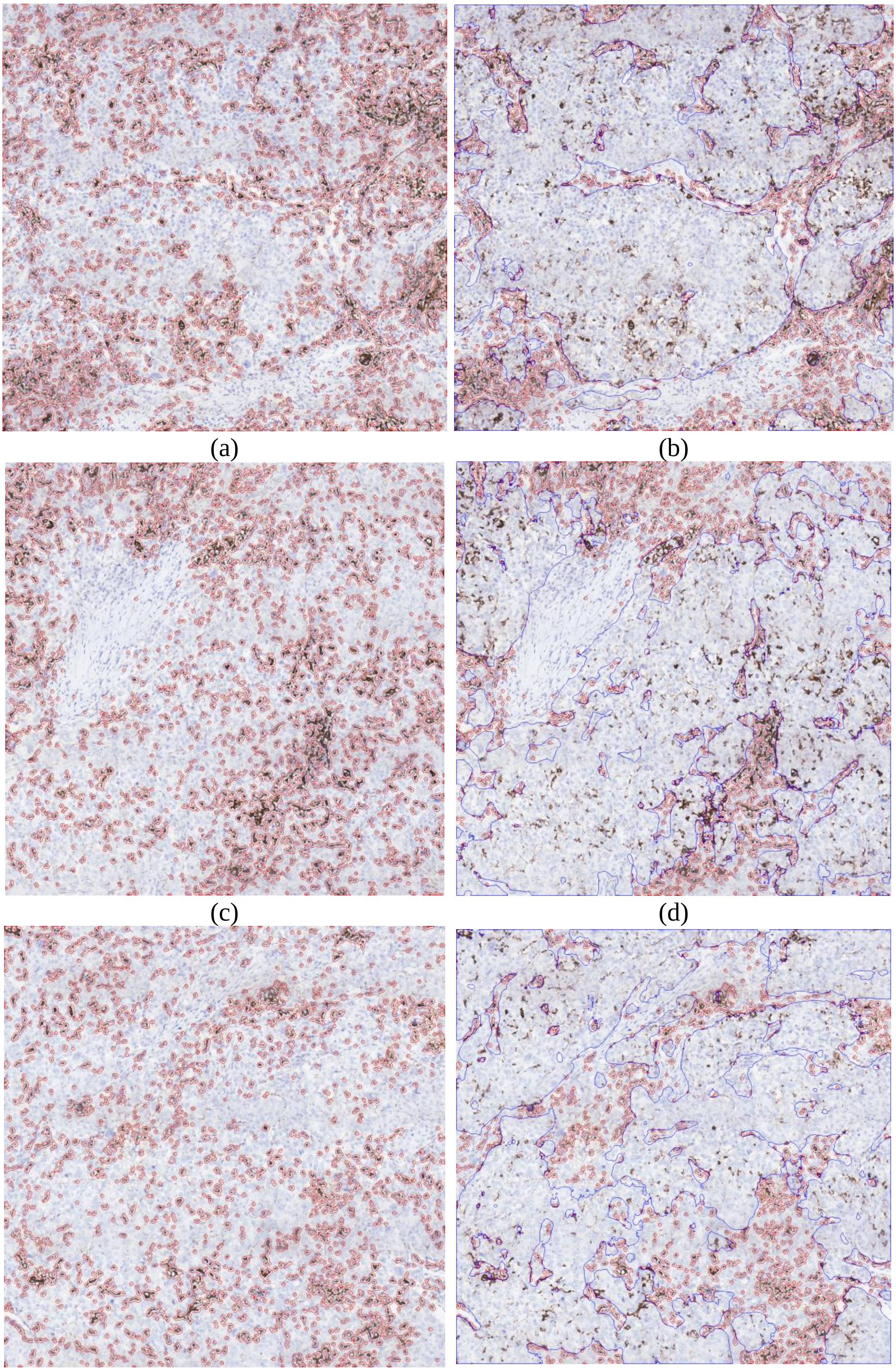

(a) (b) (c) (d) (e) (f)

Supplementary Figure 2: (a)-(b) Example 1: Ground truth IC=15.0%. (a) without epithelium/necrotic region detection, IC=32.2%. (b) with epithelium/necrotic region detection, IC=13.9%. (c)-(d) Example 2: Ground truth IC=15.0%. (c) without

epithelium/necrotic region detection, IC=28.6%. (d) with epithelium/necrotic region detection, IC=12.9%. (e)-(f) Example 3: Ground truth IC=12.0%.(e): without epithelium/necrotic region detection, IC=21.9%. (f): with epithelium/necrotic region detection, IC=11.9%.

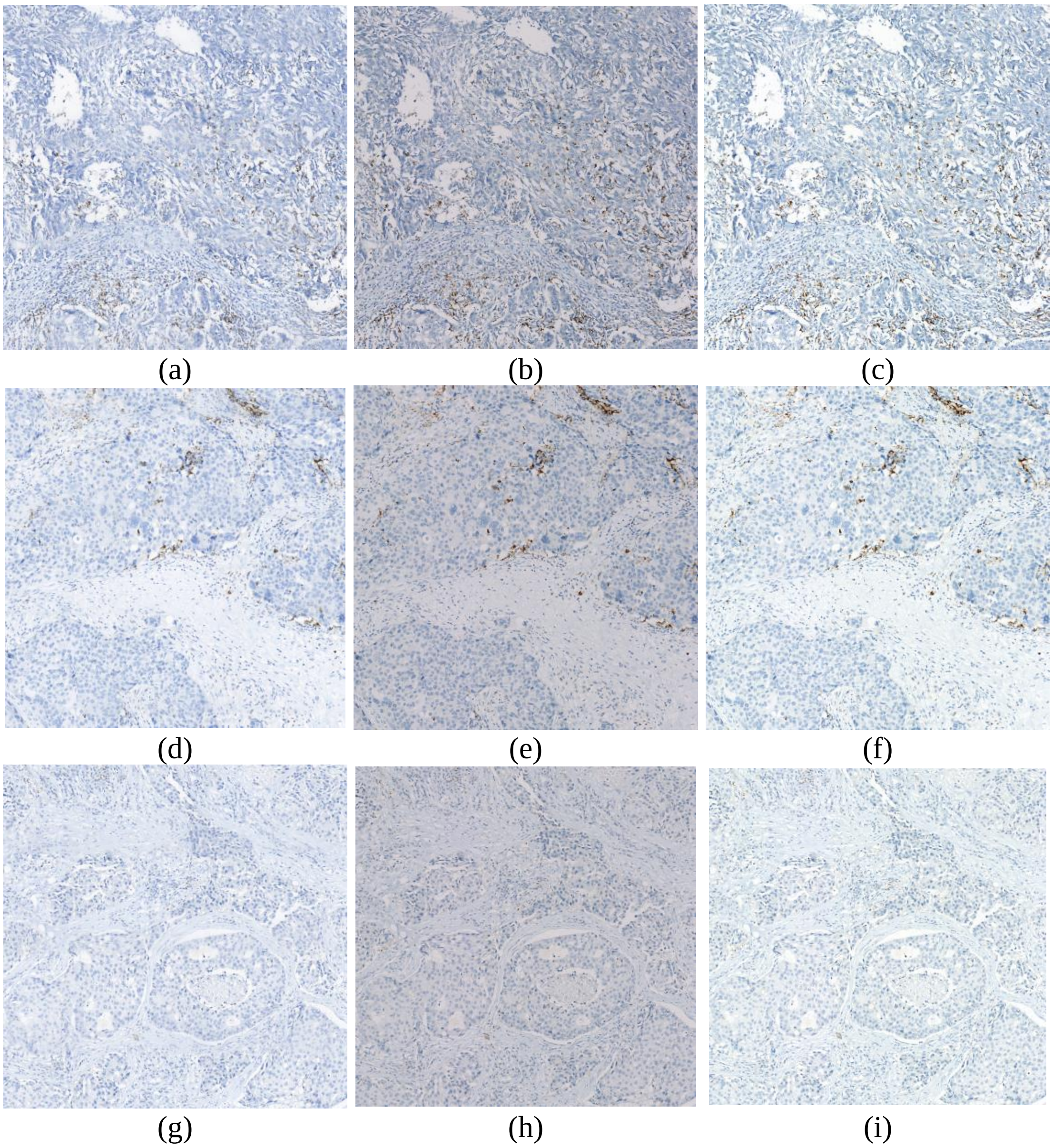

Supplementary Figure 3: Same image patches from the two slide scanners. (a)-(c) Example 1. (a) image patch from Unic scanner. IC score: AI predicted = 5.1%, ground truth = 5.0%. (b) image patch from Hamamatsu scanner. (c) image patch after white balancing from Hamamatsu scanner. IC score: AI predicted = 6.1%, ground truth = 5.0%. (d)-(f) Example 2. (d) image patch from Unic scanner. IC score: AI predicted = 2.2%, ground truth = 2.0%. (e) image patch from Hamamatsu scanner. (f) image patch after white balancing from Hamamatsu scanner. IC score: AI predicted = 2.7%, ground truth = 2.0%. (g)-(i) Example 3. (g) image patch from Unic scanner. IC score: AI predicted = 0.1%, ground truth = 0.0%. (h) image patch from Hamamatsu scanner. (i) image patch after white balancing from Hamamatsu scanner. IC score: AI predicted = 0.3%, ground truth = 0.0%.

Our experiment result shows that even binarizing at 1% IC is not a consistent task. We add an experiment to evaluate the intra-observer binary score concordance, and

note that 5.8% of scores (196 out of 3379 scores) are different for the same pathologist between RS1 and RS2 (see Supplementary Figure A8(a)). This further suggests the subjectivity of IC scoring. Therefore, even the binary scoring is a much easier job for pathologists compared to 4-category scoring or continuous scoring, computational assistance from deep learning still helps.

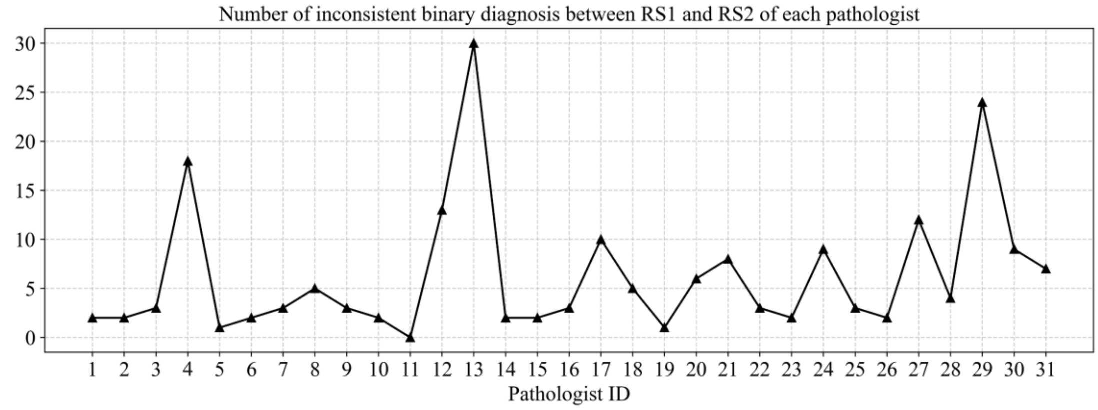

(a)

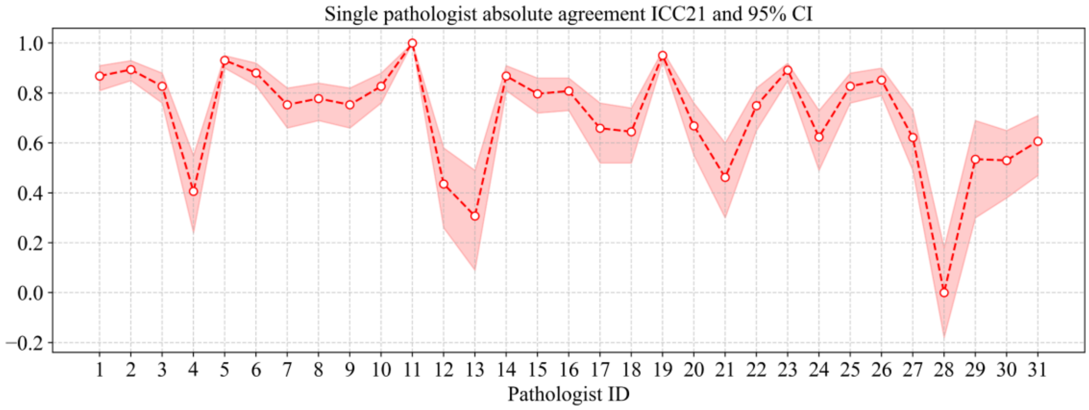

(b)

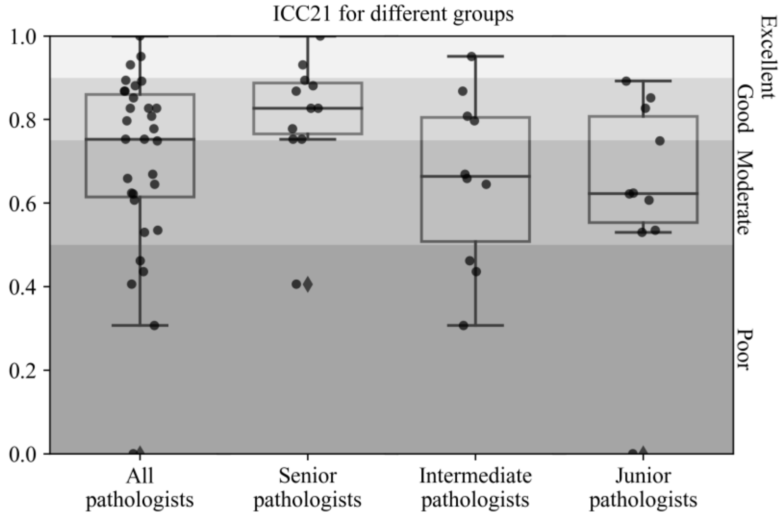

(c)

Supplementary Figure 4: Statistical of the intra-observer binary score results. (a) The number of different scoring case between RS1 and RS2 for 2-category IC score. (b) Intra-pathologist concordances ICC21 for individual pathologist between RS1 and

RS2 for 2-category IC score. Shadow area indicates the 95% CI. (c) The boxplot of Intra-pathologist concordances ICC21 for different groups of pathologists between RS1 and RS2 for 2-category IC score. The center bar of each box represents the median value, and the box body extends from the 25th to the 75th percentile of values in one group. Black circles indicate the ICC21 of individual pathologist, and black diamonds indicate the outliers.

As in Supplementary Figure 9, we calculated the intra-pathologist concordances between RS1-RS3 and RS2-RS3, respectively. The average ICC21 of RS1-RS3 was 0.756 (95% CI: 0.580-0.845), and the one of RS2-RS3 was 0.784 (95% CI: 0.642-0.858).

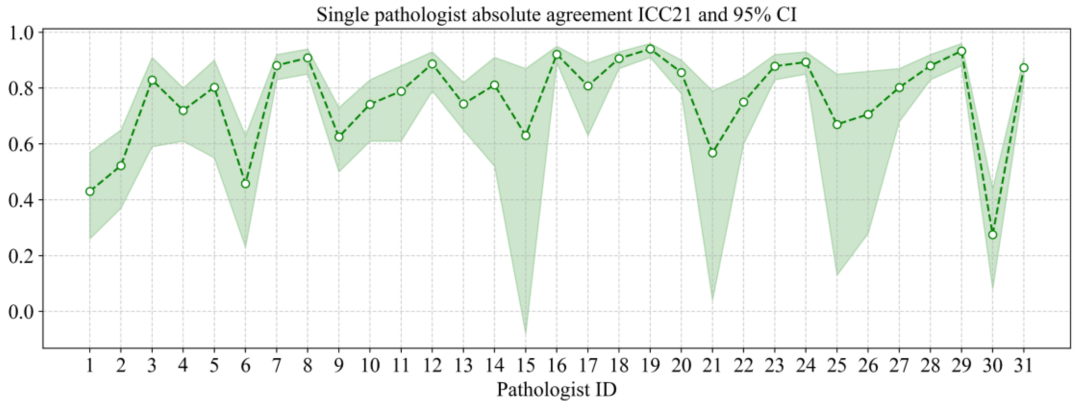

(a)

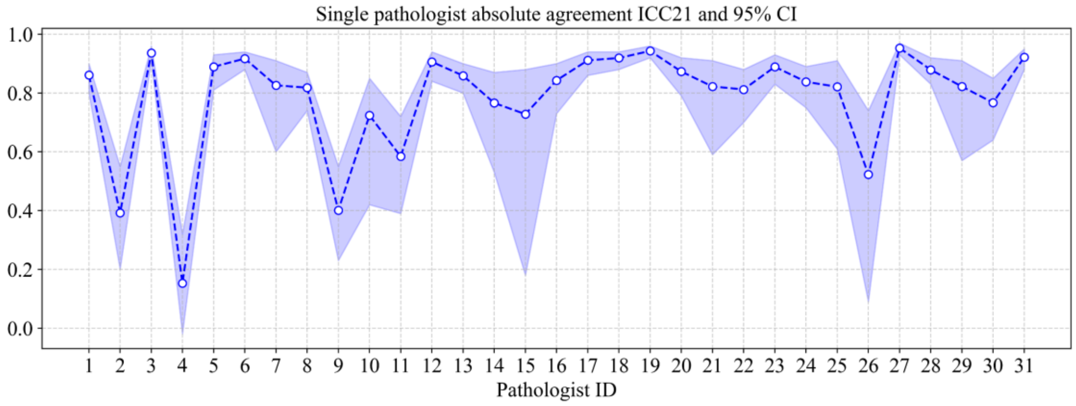

(b)

Supplementary Figure 5: Intra-pathologist concordances ICC21 and 95% CI (shadow area) for individual pathologist (a) between RS1 and RS3. The average ICC21 is 0.756 (95% CI: 0.580-0.845), and (b) between RS2 and RS3. The average ICC21 is 0.784 (95% CI: 0.642-0.858).

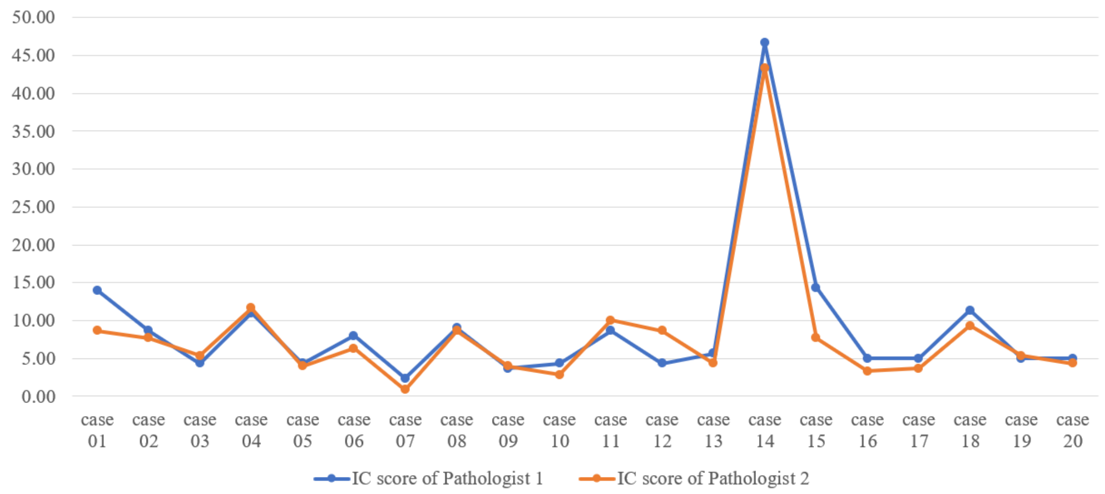

Supplementary Figure 6: Average IC scores of 20 patients by two pathologists. Three tiles are selected for each case by two pathologists independently. The IC score were the average IC scores from three tiles of the case. The ICC31 of the two pathologists scoring is 0.967 with 95% CI: 0.92-0.99.

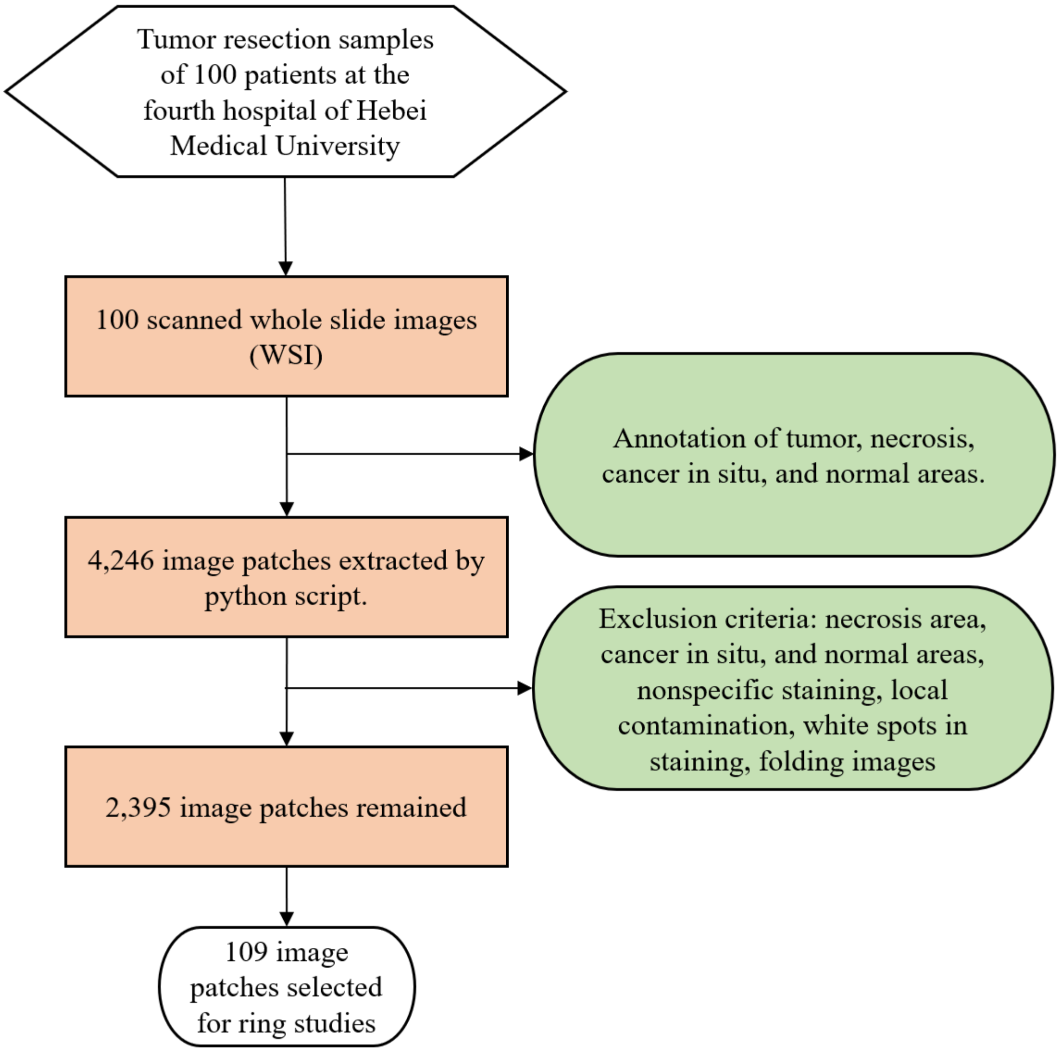

Supplementary Figure 7: STARD flow diagram of the selection of data in this study.

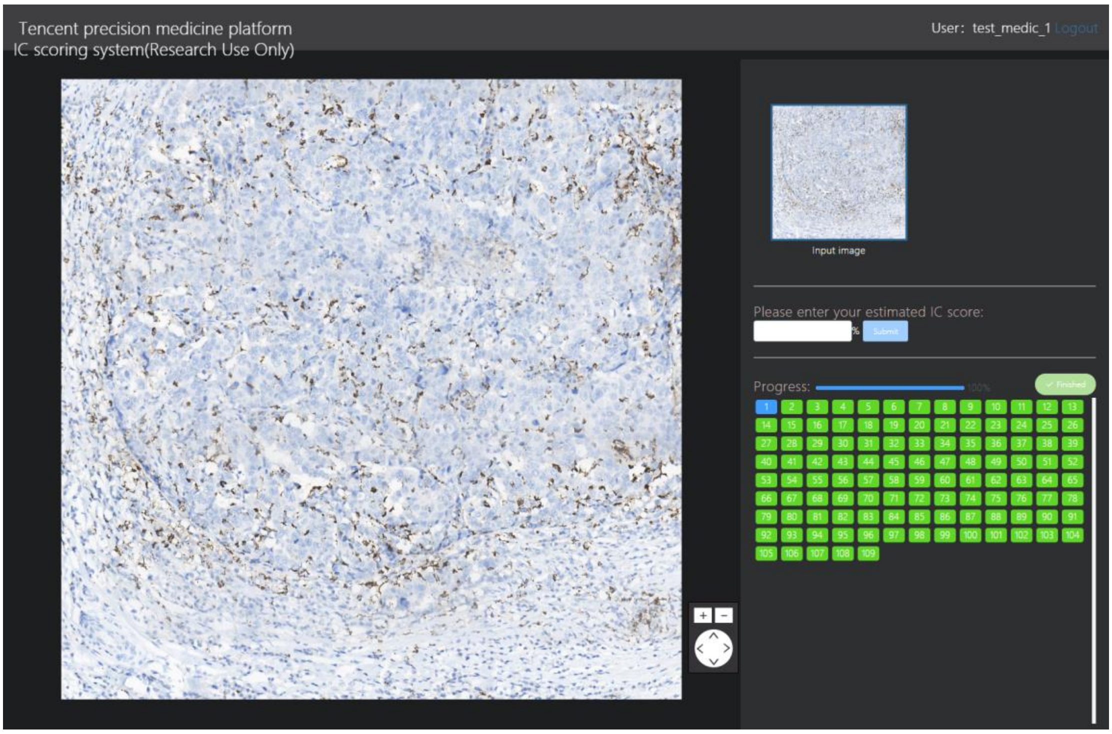

Supplementary Figure 8: Online website for PD-L1 IC scoring system in RS1and RS2.

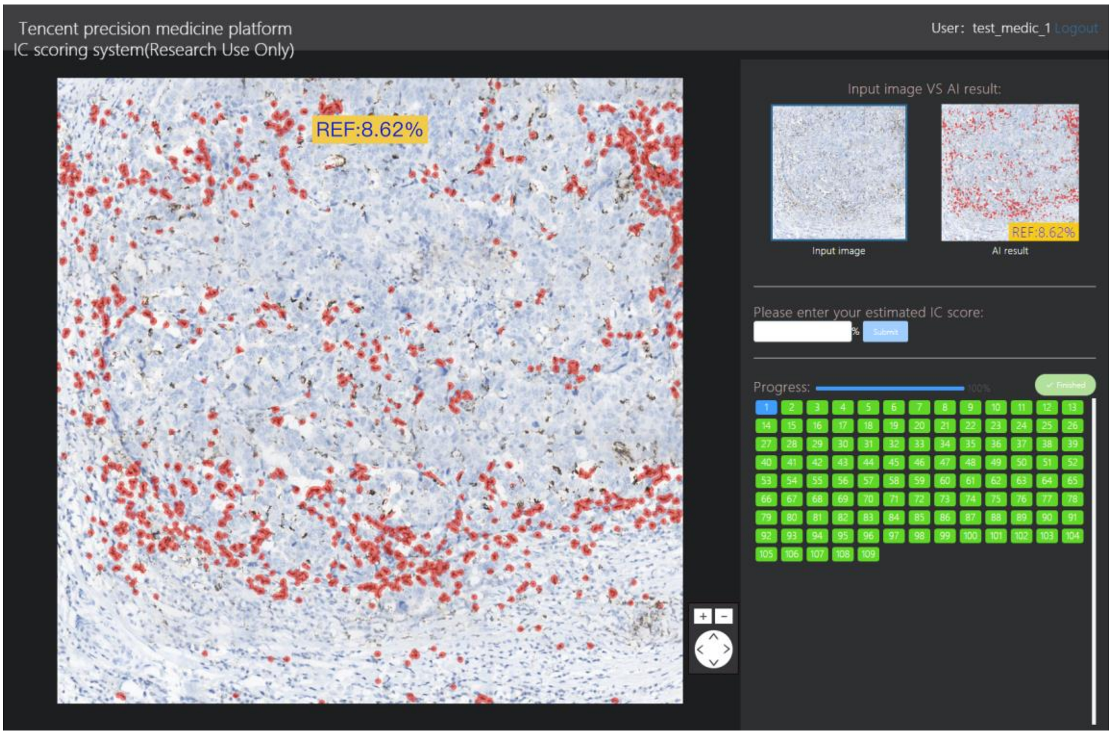

Supplementary Figure 9: Online website for PD-L1 IC scoring system with AI assisted results in RS3.

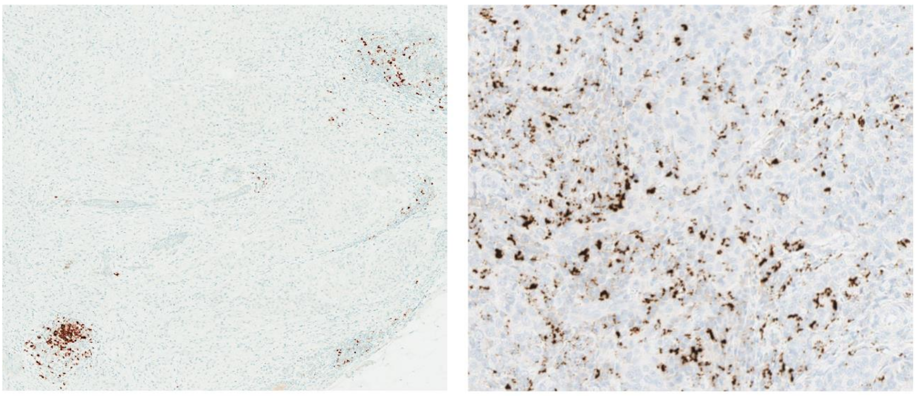

(a) (b)

Supplementary Figure 10: Roche examples for (a) aggregated case (IC >=1%) and (b) scattered case (IC >=1%) (1).

Supplementary Table 1 Scoring accuracy in Ring study.

Ring study name	pathologists	2-category score			4-category score		
		mean	std	95% CI	mean	std	95% CI
RS1	all	0.935	0.03	[0.926, 0.945]	0.679	0.11	[0.637, 0.722]
	senior	0.938	0.02	[0.928, 0.949]	0.670	0.11	[0.592, 0.747]
	intermediate	0.939	0.03	[0.919, 0.960]	0.677	0.14	[0.570, 0.784]
	junior	0.928	0.03	[0.904, 0.951]	0.692	0.08	[0.631, 0.752]
RS2	all	0.920	0.06	[0.899, 0.942]	0.710	0.12	[0.665, 0.756]
	senior	0.941	0.02	[0.925, 0.956]	0.691	0.13	[0.602, 0.781]
	intermediate	0.914	0.06	[0.871, 0.956]	0.727	0.13	[0.631, 0.822]
	junior	0.905	0.08	[0.846, 0.963]	0.715	0.11	[0.632, 0.797]
RS3	all	0.959	0.02	[0.953, 0.964]	0.815	0.03	[0.803, 0.827]
	senior	0.957	0.01	[0.947, 0.968]	0.808	0.03	[0.789, 0.827]
	intermediate	0.959	0.02	[0.943, 0.974]	0.828	0.03	[0.806, 0.849]
	junior	0.960	0.01	[0.955, 0.964]	0.809	0.04	[0.782, 0.837]

Supplementary Reference

1. Roche. VENTANA PD-L1 (SP142) Assay Interpretation Guide for Triple-Negative Breast Carcinoma (TNBC). 2019. page 1–49.
