# Supplementary material for: How can artificial intelligence models assist PD-L1 expression scoring in breast cancer: results of multi-institutional ring studies
Source: NPJ Breast Cancer. 2021 May 26;7:61. doi: 10.1038/s41523-021-00268-y (PMC8155065; doi:10.1038/s41523-021-00268-y)
Supplement: Supplementary file 2 — Reporting Summary [file 41523_2021_268_MOESM2_ESM.pdf]

nature research

Corresponding author(s): Yueping LIU

Last updated by author(s): Mar 17, 2021

Reporting Summary

Nature Research wishes to improve the reproducibility of the work that we publish. This form provides structure for consistency and transparency in reporting. For further information on Nature Research policies, see our Editorial Policies and the Editorial Policy Checklist.

Statistics

For all statistical analyses, confirm that the following items are present in the figure legend, table legend, main text, or Methods section.

n/a	Confirmed	
☐	☒	The exact sample size (*n*) for each experimental group/condition, given as a discrete number and unit of measurement
☐	☒	A statement on whether measurements were taken from distinct samples or whether the same sample was measured repeatedly
☐	☒	The statistical test(s) used AND whether they are one- or two-sided<br>*Only common tests should be described solely by name; describe more complex techniques in the Methods section.*
☐	☒	A description of all covariates tested
☐	☒	A description of any assumptions or corrections, such as tests of normality and adjustment for multiple comparisons
☐	☒	A full description of the statistical parameters including central tendency (e.g. means) or other basic estimates (e.g. regression coefficient) AND variation (e.g. standard deviation) or associated estimates of uncertainty (e.g. confidence intervals)
☐	☒	For null hypothesis testing, the test statistic (e.g. *F*, *t*, *r*) with confidence intervals, effect sizes, degrees of freedom and *P* value noted<br>*Give P values as exact values whenever suitable.*
☒	☐	For Bayesian analysis, information on the choice of priors and Markov chain Monte Carlo settings
☒	☐	For hierarchical and complex designs, identification of the appropriate level for tests and full reporting of outcomes
☐	☒	Estimates of effect sizes (e.g. Cohen's *d*, Pearson's *r*), indicating how they were calculated

Our web collection on statistics for biologists contains articles on many of the points above.

Software and code

Policy information about availability of computer code

Data collection	The slides were scanned to WSI files by using the software of Unic digital scanner precision 600 Series, and Hamamatsu NanoZoomer S210 Digital slide scanner C13239-01. The image tiles were extracted from the WSI files by software Python 3.6.5 with mainly the package Openslide (Ver 1.1.1), and Numpy (Ver 1.15.0).
Data analysis	The data analysis were used Microsoft Office 365 Excel, Python 3.6.5 with the Scikit-learn (Ver 0.23.1), Pingouin (Ver 0.3.3) packages, R language (RStudio Ver 1.3.959) with the readxl, tidyverse, and DescTools packages.

For manuscripts utilizing custom algorithms or software that are central to the research but not yet described in published literature, software must be made available to editors and reviewers. We strongly encourage code deposition in a community repository (e.g. GitHub). See the Nature Research guidelines for submitting code & software for further information.

Data

Policy information about availability of data

All manuscripts must include a data availability statement. This statement should provide the following information, where applicable:

- Accession codes, unique identifiers, or web links for publicly available datasets
- A list of figures that have associated raw data
- A description of any restrictions on data availability

The data and code for analysis and experiments were publicly available here :
https://github.com/LWCHN/PDL1_SP142_Breast_IC_score
The code and data can be used only for "non-commercial" purpose and under the permission of the correspond author.

Field-specific reporting

Please select the one below that is the best fit for your research. If you are not sure, read the appropriate sections before making your selection.

☒ Life sciences ☐ Behavioural & social sciences ☐ Ecological, evolutionary & environmental sciences

For a reference copy of the document with all sections, see nature.com/documents/nr-reporting-summary-flat.pdf

Life sciences study design

All studies must disclose on these points even when the disclosure is negative.

Sample size	One hundred tumor resection samples (formalin-fixed, paraffin-embedded blocks)from 100 patients with invasive breast cancer were collected in the fourth hospital of Hebei Medical University from January to June 2019.All immunostained slides were scanned using the Unic digital scanner (precision 600 Series, Unic Technologies.Firstly,from the scanned whole slide images (WSI), two pathologists manually identified the tumor regions. They also annotated the necrosis area, cancer in situ, and normal areas by strictly following the scoring guideline of Ventana PD-L1 (SP142) in breast cancer provided by Roche guide . Secondly, from the tumor regions, sliding windows with no overlap were scanned through the tumor regions and generated 4,246 image patches. After that, images with manually identified necrosis area, cancer in situ, and normal areas were excluded and 2,395 image patches remained. Then, considering the workload, 109 image patches were randomly proposed from the 2,395 image patches, with the criteria that the proposed patches should not have non-specific staining, focal contamination, and folding, and should not be similar to other patches in the set. At last, the selected 109 image patches were used for this study.
Data exclusions	From the 100 scanned whole slide images (WSI), two pathologists manually identified the tumor regions. They also annotated the necrosis area, cancer in situ, and normal areas by strictly following the scoring guideline of Ventana PD-L1 (SP142) in breast cancer provided by Roche guide . Secondly, from the tumor regions, sliding windows with no overlap were scanned through the tumor regions and generated 4,246 image patches. After that, images with manually identified necrosis area, cancer in situ, and normal areas were excluded and 2,395 image patches remained. 109 image patches were randomly proposed from the 2,395 image patches, with the criteria that the proposed patches should not have non-specific staining, focal contamination, and folding, and should not be similar to other patches in the set.
Replication	From the repeated ring study 1 and ring study 2, it can be found that the concordance results in each respective ring study was similar. The ICC31 was 0.674 (95% confidence interval (CI): 0.614-0.735) for RS1, and 0.736 (95% CI: 0.683-0.789) for RS2. Both values were less than 0.75, and was hence interpreted as "moderate" concordance.
Randomization	The participants were divided into three groups according to their experience: senior (≥10 years, 11 pathologists), intermediate (≥5 years but <10 years, 10 pathologists), and junior (≥2 years but <5 years, 10 pathologists). The results of each group were discussed in the study.
Blinding	During the ring studies, each participant was independently logged onto an online website, viewed the images, estimated the area of stained ICs, provided the IC score, and entered their assessments. The participants were blind to each other.

Reporting for specific materials, systems and methods

We require information from authors about some types of materials, experimental systems and methods used in many studies. Here, indicate whether each material, system or method listed is relevant to your study. If you are not sure if a list item applies to your research, read the appropriate section before selecting a response.

Materials & experimental systems

n/a	Involved in the study	
☐	☒	Antibodies
☒	☐	Eukaryotic cell lines
☒	☐	Palaeontology and archaeology
☒	☐	Animals and other organisms
☐	☒	Human research participants
☒	☐	Clinical data
☒	☐	Dual use research of concern

Methods

n/a	Involved in the study	
☒	☐	ChIP-seq
☒	☐	Flow cytometry
☒	☐	MRI-based neuroimaging

Antibodies

Antibodies used	PD-L1 (clone SP142, Ventana Medical Systems, Tucson, USA)
Validation	According to the VENTANA PD-L1 (SP142) Assay instructions by Roche,the verification steps were as follows:on benchmark XT automatic immunohistochemistry, set the CC1 Cell Conditioning to 48 minutes,rabbit monoclonal negative control and tonsil tissue control were selected,primary antibody to16 minutes,amplification multimer for 8 minutes,hematoxylin II and bluing reagent for 4 minutes. Manufacturer's website:www.ventana.com. citations:1.Anatomic Pathology Checklist. College of American Pathologists. Jul

28, 2015.   2.CLSI. Quality assurance for design control and implementation of immunohistochemistry assay: approved guidelines. 2nd edition Wayne, PA, USA:Clinical and Laboratory Standards Institute; 2011.

Human research participants

Policy information about studies involving human research participants

Population characteristics	Among the 100 patients, 39 patients under 50 years old, 61 patients over 50 years old. 97 patients were diagnosed with Invasive carcinoma of no special type, 2 were diagnosed with invasive lobular carcinoma and 1 was diagnosed with meta-plastic. 3 cases were classified as grade 1, 37 cases as grade 2 and 60 cases as grade 3. carcinoma. 28 cases were clinical stage 1, 57 cases were stage 2 and 15 cases were stage 3.
Recruitment	100 patients were surgically diagnosed as invasive breast cancer in the fourth hospital of Hebei Medical University from January to June 2019. All patients did not receive any treatment before operation.One hundred tumor resection samples (formalin-fixed, paraffin-embedded blocks) were collected from the 100 patients.
Ethics oversight	All tissues and data were retrieved under the permission of the institutional research ethics board of the Fourth Hospital of Hebei Medical University with the declaration number of 2020KY112.

Note that full information on the approval of the study protocol must also be provided in the manuscript.
